# Self-immolative nanosensitizer for glutathione depletion- assisted sonodynamic therapy

**DOI:** 10.7150/thno.75007

**Published:** 2022-10-24

**Authors:** Chan Ho Kim, Dong Gil You, Pramod Kumar E. K., Kyung Hee Han, Wooram Um, Jeongjin Lee, Jae Ah Lee, Jae Min Jung, Heegun Kang, Jae Hyung Park

**Affiliations:** 1 School of Chemical Engineering, College of Engineering, Sungkyunkwan University, 2066 Seobu-ro, Jangan-gu, Suwon 16419, Republic of Korea.; 2 Department of Health Sciences and Technology, SAIHST, Sungkyunkwan University, 81 Irwon-ro, Gangnam-gu, Seoul, 06351 Republic of Korea.; 3 Biomedical Institute for Convergence at SKKU (BICS), Sungkyunkwan University, 2066 Seobu-ro, Jangan-gu, Suwon 16419, Republic of Korea.

**Keywords:** self-immolative polymer, TiO_2_ nanoparticles, reactive oxygen species, glutathione, sonodynamic therapy

## Abstract

**Background:** Despite remarkable advances in sonodynamic therapy (SDT) of cancer, the low reactive oxygen species (ROS) quantum yield of the sonosensitizer remains a critical concern in glutathione (GSH)-overexpressing cancer cells.

**Methods:** For enhanced SDT, we report hydrophilized self-immolative polymer (SIP)-decorated TiO_2_ nanoparticles (HSIPT-NPs) to achieve on-demand GSH depletion and ROS generation.

**Results:** Upon intracellular delivery of HSIPT-NPs into hydrogen peroxide-rich cancer cells, SIP is degraded through electron transfer to produce GSH-depleting quinone methide, reprogramming GSH*^high^* cancer cells into GSH*^low^* phenotype. In the presence of ultrasound, compared to conventional TiO_2_ NPs, HSIPT-NPs induce significantly higher oxidative stress to cancer cells by incapacitating their antioxidant effects. SDT with HSIPT-NPs effectively inhibit tumor growth in mice via the synergistic effects of GSH depletion and ROS generation.

**Conclusion:** On the basis of their ability to reprogram cancer cells, HSIPT-NPs offer considerable potential as a nanosensitizer for enhanced SDT.

## Introduction

According to the medical pursuit of non- or minimally invasive surgery, ultrasound-mediated approaches have gained attention as alternatives to treat cancer 1, 2. In particular, sonodynamic therapy (SDT), which generates cytotoxic reactive oxygen species (ROS) by ultrasonic excitation of a sonosensitizer to kill cancer cells, has been extensively investigated in pre-clinical studies of deeply located tumors with precise margin control 3. However, SDT is only considered partially successful because it relies highly on the performance of conventional sonosensitizers with low ROS quantum yields, such as titanium dioxide nanoparticles (TiO_2_ NPs) 4, 5. Geared by recent advances in nanobiotechnology, various nanosensitizers have been rationally designed and have significantly improved the efficacy of SDT 6-8. For instance, approaches that prevent electron-hole recombination or increase the absorption spectrum by combining TiO_2_ NPs with noble metals such as Pt, Au, and Ag have been developed 5, 9, 10. However, from the perspective of the SDT mechanism, low ROS generation efficiency is inevitable because the sonoluminescence generated by ultrasound poorly activates the nanosensitizer 11.

The tumor microenvironment (TME) has unique features, such as high ROS levels, low pH, hypoxia, and high intracellular glutathione (GSH) levels 12-15. Notably, the overexpression of GSH fosters favorable environments to protect cancer cells from oxidative stress by ROS, suggesting that the therapeutic efficacy of SDT could be largely compromised in GSH-overexpressing cancer cells 16, 17. Therefore, many efforts have been made to investigate strategies to reduce GSH levels in cancer cells 18-21. For example, 1,4-quinone methide (QM), an antioxidant inhibitor, rapidly alkylates GSH and triggers apoptotic cell death 20. However, because QM is readily converted into 4-hydroxybenzyl alcohol in the presence of water, a large amount of QM should be delivered at the intracellular level to react effectively with GSH 22. Therefore, to minimize side effects on normal cells and maximize therapeutic efficacy for cancer, it is necessary to develop tumor-specific QM delivery systems.

**Figure 1:**
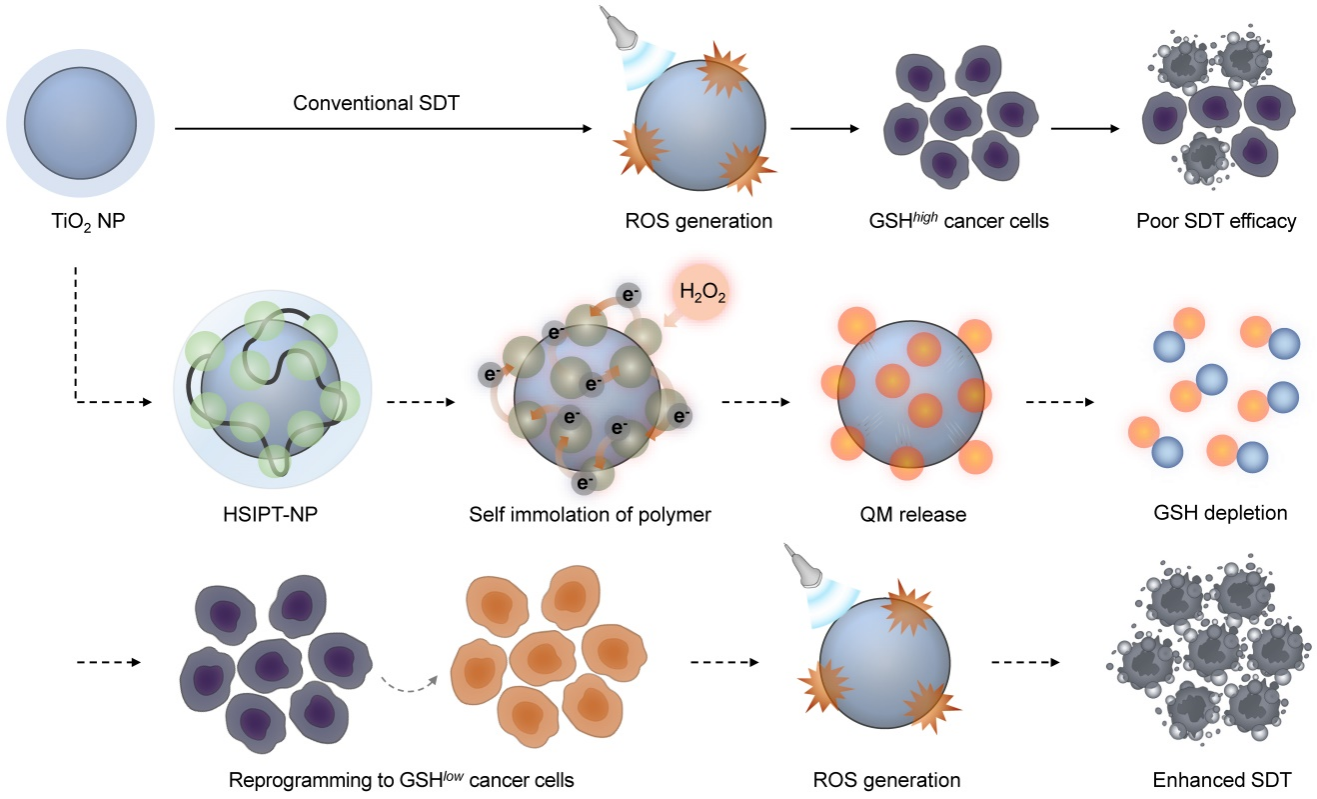
Schematic illustration of HSIPT NPs for enhanced SDT. After intracellular delivery of HSIPT-NPs into the hydrogen peroxide-rich cancer cells, SIP on the surface of TiO_2_ NPs are rapidly degraded through electron transfer, resulting in QM-mediated GSH depletion. Owing to their unique nature of reprogramming GSH*^high^* cancer cells to GSH*^low^* phenotype, HSIPT-NPs enhance the anti-tumor efficacy of SDT.

A self-immolative polymer (SIP) is a macromolecule that is rapidly depolymerized into a small molecule through the domino-like cleavage of the backbone or terminal in a specific stimulus 23. Owing to its unique characteristics, SIP has gained considerable attention in the field of drug delivery systems 24-28. Notably, in response to a specific stimulus, SIP can rapidly and precisely deliver a large amount of therapeutics to the desired site 26. However, there have been no previous studies to improve the antitumor efficacy of SDT in combination with SIP. In this study, for the first time, we designed hydrophilized SIP-decorated TiO_2_ nanoparticles (HSIPT-NPs) for enhanced SDT (Figure 1). After intracellular delivery of HSIPT-NPs to hydrogen peroxide-rich cancer cells, SIP on the surface of TiO_2_ NPs can rapidly immolate through electron transfer, resulting in cell death by QM-mediated GSH depletion in an oxygen-independent manner. In addition, HSIPT-NPs generate a large quantity of cytotoxic ROS through synergistic oxidative stress when exposed to ultrasound. On the basis of unique nature of reprogramming GSH*^high^* cancer cells to the GSH*^low^* phenotype, the SIP-based nanosensitizer offers considerable potential for enhanced SDT.

## Materials and methods

### Materials

2-Aminoethyl dihydrogen phosphate (AEP) (98%) was purchased from Tokyo Chemical Industry (TCI, Tokyo, Japan); DAEMA (98%), copper (I) bromide, 2,2-bipyridyl (99%), carboxymethyl-dextran sodium salt (10-20 kDa), titanium(IV) oxide (anatase, <25 nm particle size, 99.7%), α-bromoisobutyryl bromide (98%), mPBA, methylthiazolyldiphenyl-tetrazolium bromide (MTT), DPBF, thiol tracker violet, and 2,7-dichlorofluorescin diacetate (DCF-DA) were purchased from Sigma-Aldrich (St. Louis, MO, USA). All other chemicals were of analytical grade and used without further purification. SCC7 and L929 cells were purchased from the American Type Culture Collection (Manassas, MD, USA). For cell culture, RPMI 1640 and fetal bovine serum (FBS) were purchased from Capricorn Scientific (Ebsdorfergrund, Germany). The antibiotic-antimycotic solution, trypsin-EDTA, and Dulbecco's phosphate-buffered saline (DPBS) were obtained from Welgene (Daegu, Korea). All experiments involving live animals were carried out in accordance with the relevant laws and institutional guidelines of Sungkyunkwan University (SKKUIACUC2019-08-22-1).

### Synthesis of TiO_2_-AEP-Br

First, 500 mg of TiO_2_ NPs were added to 100 mL formamide and the mixture was sonicated for 15 min. Then, 1 mL of AEP solution (50 mg/mL in deionized water) was slowly added and vigorously stirred for 6 h at 25 °C. To remove unreacted chemicals, the resulting solution was dialyzed against distilled water for 3 days using a membrane tube (molecular weight cut-off = 12 kDa, Spectrum Laboratories Inc., CA, USA), followed by lyophilization. Next, 200 mg of AEP-decorated TiO_2_ NPs (TiO_2_-AEP) was dispersed in 15 mL of dichloromethane and 1,100 mg of triethylamine was added, followed by mixing the solution via stirring for 40 min at 4 °C. To substitute the amine group of TiO_2_-AEP for the bromo group, 20 mL of α-bromoisobutyryl bromide solution (75 mg/mL in dichloromethane) was added dropwise to the mixture, and the resulting solution was sequentially stirred at 4 °C for 4 h and 25 °C for another 12 h. Then, the resulting product was washed three times with an excess amount of water/acetone (1/1, v/v) and dried under vacuum for 48 h at 25 °C to obtain TiO_2_-AEP-Br.

### Synthesis of TiO_2_-P(DAEMA)

TiO_2_-P(DAEMA) was synthesized via atom transfer radical polymerization. In brief, TiO_2_-AEP-Br (200 mg), DAEMA (2.1 mL), and 2,2-bipyridyl (39 mg) were added to a Schlenk flask and mixed with 6 mL of dimethylformamide water/isopropanol (1/1/1, v/v/v). After the mixture solution was purged with N_2_ for 30 min, 18 mg of copper bromide was added to the mixture solution, and the reaction was conducted at 25 °C for 24 h under N_2_. Subsequently, the mixture was thoroughly washed five times with dimethylformamide/methanol (1/1, v/v) and re-suspended in dimethylformamide. The resulting solution was dialyzed against distilled water for 3 days using a dialysis tube (molecular weight cut-off = 12 kDa), followed by lyophilization to obtain TiO_2_-P(DAEMA).

### Synthesis of HSIPT-NPs

First, SIPT-NP was prepared through a coupling reaction between the tertiary amine group of TiO_2_-P(DAEMA) and the brome group of mPBA. In brief, 100 mg of TiO_2_-P(DAEMA) was dispersed in 30 mL of dimethylformamide. Then, 184 mg of mPBA was added to the mixture solution, followed by stirring for 48 h at 45 °C. The reaction mixture was sequentially dialyzed against methanol/distilled water (1v/1v-1v/3v) for 3 days and against distilled water for another 2 days using a dialysis tube (molecular weight cut-off = 50 kDa). The resulting solution was centrifuged at 350 × g for 15 min to remove large aggregates. Finally, the supernatant was filtered using a syringe filter with 0.8 µm pore size, and the filtered solution was lyophilized to obtain SIPT-NP. Next, 20 mg of SIPT-NP was dispersed in 20 mL of distilled water. Subsequently, the reaction solution was added dropwise into 20 mL of CMD solution (40 mg/mL in distilled water), followed by stirring for 6 h at 25 °C. The reaction solution was dialyzed (molecular weight cut-off = 50 kDa) against distilled water for 4 days, followed by lyophilization. In addition, as a control sample, we prepared HT-NPs (without SIP) following procedures previously described in a published article 4.

For *in vitro* and *in vivo* studies, HSIPT-NPs were labeled with Flamma 675 dichlorotriazine (Bioacts, Incheon, Korea). Briefly, 10 mg of HSIPT-NPs was dispersed in 2 mL of distilled water, followed by the addition of 15 µL of fluorophore stock solution (1 mg/mL in DMSO). After 12 h of stirring, the resulting solution was dialyzed against distilled water for 3 days using a dialysis tube (molecular weight cut-off = 3.5 kDa) and freeze-dried.

### Characterization

The chemical structures of SIP on the surface of TiO_2_ NPs and HSIPT-NPs were analyzed using ^1^H NMR (500 MHz, Bruker, Billerica, MA, USA) and FTIR (IFS-66/S, Bruker, Billerica, MA, USA). The hydrodynamic size and zeta potential of HSIPT-NPs were measured using a Zetasizer (Nano ZS90, Malvern Instruments, Malvern, UK) with a He-Ne laser (633 nm) at 90° collecting optics. For stability test, we prepared SIPT-NPs and HSIPT-NPs (1 mg/mL) solutions in PBS (pH 7.4) and measured their hydrodynamic size and zeta potential for predetermined times. The morphology and crystal structure of the HSIPT-NPs were measured using TEM (JEM-2100F, JEOL, Japan). In addition, EDS mapping images were obtained using EDS-TEM at 200 kV (JEM-2100F, JEOL, Tokyo, Japan).

### Cell culture

For SCC7 (a murine squamous cell carcinoma) and L929 (a murine fibroblast cell line), we used RPMI 1640 with 10% FBS and 1% antibiotic-antimycotic solution, and the cells were maintained at 37 °C in a humidified CO_2_ incubator.

### *In vitro* GSH depletion test

To observe GSH depletion using confocal imaging, SCC7 and L929 cells were seeded in 35-mm confocal dishes at a density of 3 × 10^5^ cells. After 24 h, the cells were incubated with HT-NPs and HSIPT-NPs (0.2-2 µg/mL of Ti) at 37 °C for 24 h. The cells were then washed twice with DPBS (pH 7.4) and incubated with 0.2 mL of thiol tracker (40 µM, T10095, Invitrogen, Waltham, MA, USA) at 37 °C for 30 min. After washing twice with DPBS, the cells were fixed with 4% paraformaldehyde solution. Then, the cells were observed using a confocal laser microscope (TCS SP8 HyVolution, Leica Microsystems CMS GmbH, Wetzlar, Germany) in the BIORP of Korea Basic Science Institute (KBSI).

To analyze intracellular GSH levels using flow cytometry, SCC7 and L929 cells were seeded at a density of 1 × 10^6^ cells in a 100 mm dish and incubated for 24 h. Then, the cells were treated with a serum-free medium containing HT-NPs or HSIPT-NPs (2 μg/mL of Ti) for 24 h. After washing twice with DPBS, the cells were stained with an intracellular GSH detection assay kit (Abcam, ab112132, Cambridge, UK), according to the manufacturer's instructions. The fluorescence intensity was measured using flow cytometry (Guava EasyCyte, Merck Millipore, Burlington, MA, USA).

### MTT assay

To investigate the cytotoxicity of HT-NPs and HSIPT-NPs, SCC7 and L929 cells were seeded onto 96-well plates at a density of 1 × 10^4^ cells and incubated for 24 h. Then, the cell media were washed with DPBS, replaced with serum-free media containing HT-NPs or HSIPT-NPs (0.2-2 µg/mL of Ti), and incubated for 24 h. Then, the cell viability was determined using an MTT assay. The absorbance was measured at 570 nm using a microplate reader (VersaMax, Molecular Devices, San Joes, CA, USA).

To further investigate the cytotoxicity of HT-NPs and HSIPT-NPs under hypoxic conditions, SCC7 cells were seeded onto 96-well plates at a density of 1 × 10^4^ cells and incubated in 95% N_2_ and 5% CO_2_ atmosphere for 24 h. After washing the cells twice with DPBS, the cells were treated with HT-NPs or HSIPT-NPs (2 µg/mL of Ti) and incubated for 24 h. Then, the MTT assay was performed identically, as described earlier.

### *In vitro* ROS generation

To measure the generated ROS levels, HT-NPs and HSIPT-NPs (6 µg /mL of Ti) were dispersed in 1 mL of distilled water and dimethylformamide (1v/3v) containing 40 µM of DPBF. Then, 1 mL of the mixture solution was placed in a 3% agarose mold (0.8 cm in pore diameter), followed by irradiation with US (VIFU2000, Alpinion, Anyang-si, Korea) at three points for 100 s/point (power: 5 W, duty cycle: 50%, pulse repetition frequency: 1 Hz, XY interval: 2 mm). The total ultrasound irradiation time was 20 min. The absorbance of DPBF was measured at 415 nm using a UV-vis spectrometer (Optizen 3220 UV, Mecasys Co, Daejeoun, Korea).

### Intracellular ROS generation

To observe intracellular ROS generation, SCC7 cells were seeded in 35-mm confocal dishes at a density of 3 × 10^5^ cells. Afterward, the cell media were washed with DPBS, replaced with serum-free media containing HT-NPs or HSIPT-NPs (2 µg/mL of Ti), and incubated for 24 h. The cell suspension was exposed to US (power: 5 W, duty cycle: 50%, pulse repetition frequency: 1 Hz, XY interval: 2 mm) at three points for 100 s/point and incubated with DCF-DA (20 µM) for 20 min 29. After nuclei staining with Hoechst, the cells were fixed and imaged identically using a confocal laser microscope, as described earlier.

To further investigate the intracellular ROS, SCC7 cells were seeded at a density of 1 × 10^6^ cells in a 100 mm dish and incubated for 24 h. Then, the cells were treated with HT-NPs or HSIPT-NPs (2 µg/mL of Ti) for 24 h, exposed to US, and identically stained with 20 µM DCF-DA, as described earlier. After washing twice with DPBS, the cells were incubated with FACS buffer (1% FBS containing PBS, pH 7.4) and analyzed using flow cytometry. Fluorescence intensities were quantified using the FlowJoTM software (BD Life Science, Franklin Lakes, NJ, USA).

### *In vitro* sonotoxicity assay

To investigate cytotoxicity by SDT *in vitro*, SCC7 cells were seeded at a density of 1 × 10^6^ cells in a 100 mm dish and incubated for 24 h. Then, the cells were treated with HT-NPs or HSIPT-NPs (0.4 µg/mL of Ti) for 24 h. In this experiment, to verify the synergistic effects of ROS generation and GSH depletion, a low concentration of HSIPT-NPs (0.4 μg/ml of Ti) was chosen to treat cancer cells. Then, the cell suspension was filled into a 3% agarose mold and exposed to US (power: 5 W, duty cycle: 50%, pulse repetition frequency: 1 Hz, XY interval: 2 mm) at three points for 100 s/point. Cell viability was determined identically using an MTT assay as described earlier.

### *In vivo* antitumor efficacy

First, SCC7 cells (1 × 10^6^ cells/head) were injected into the left flank of C3H/HeN mice (5 weeks old, male). When the tumor volume reached 50-100 mm^3^, mice were divided into six groups: (i) PBS, (ii) PBS + US, (iii) HT-NP, (iv) HT-NP + US, (v) HSIPT-NP, and (vi) HSIPT-NP + US. Each sample (Ti 2 mg/kg) was intravenously injected into SCC7 tumor-bearing mice. At 12 h after injection, the tumor regions were treated with US (power: 10 W, duty cycle: 20%, pulse repetition frequency: 1 Hz, XY interval, 2 mm). Each treatment was administered a total of seven times, once every 3 days. The tumor volume was calculated as the largest diameter × smallest diameter^2^ × 0.5 and recorded along with the bodyweight every day. Tumor tissues were collected on day 29 and fixed in 4% paraformaldehyde. Then, the tissues were embedded in paraffin and sectioned into 6-µm thick sections on glass slides. After hematoxylin and eosin (H&E) staining, the tissues were observed using a microscope slide scanner (Axio Scan.Z1, Carl Zeiss, Oberkochen, Germany).

### Statistical analysis

All values are expressed as the mean ± SD. Statistically significant differences among the groups were analyzed using t-test or One-Way ANOVA. Differences with P < 0.05 were considered statistically significant.

## Results and Discussion

### Characterization of HSIPT-NPs

**Figure 2:**
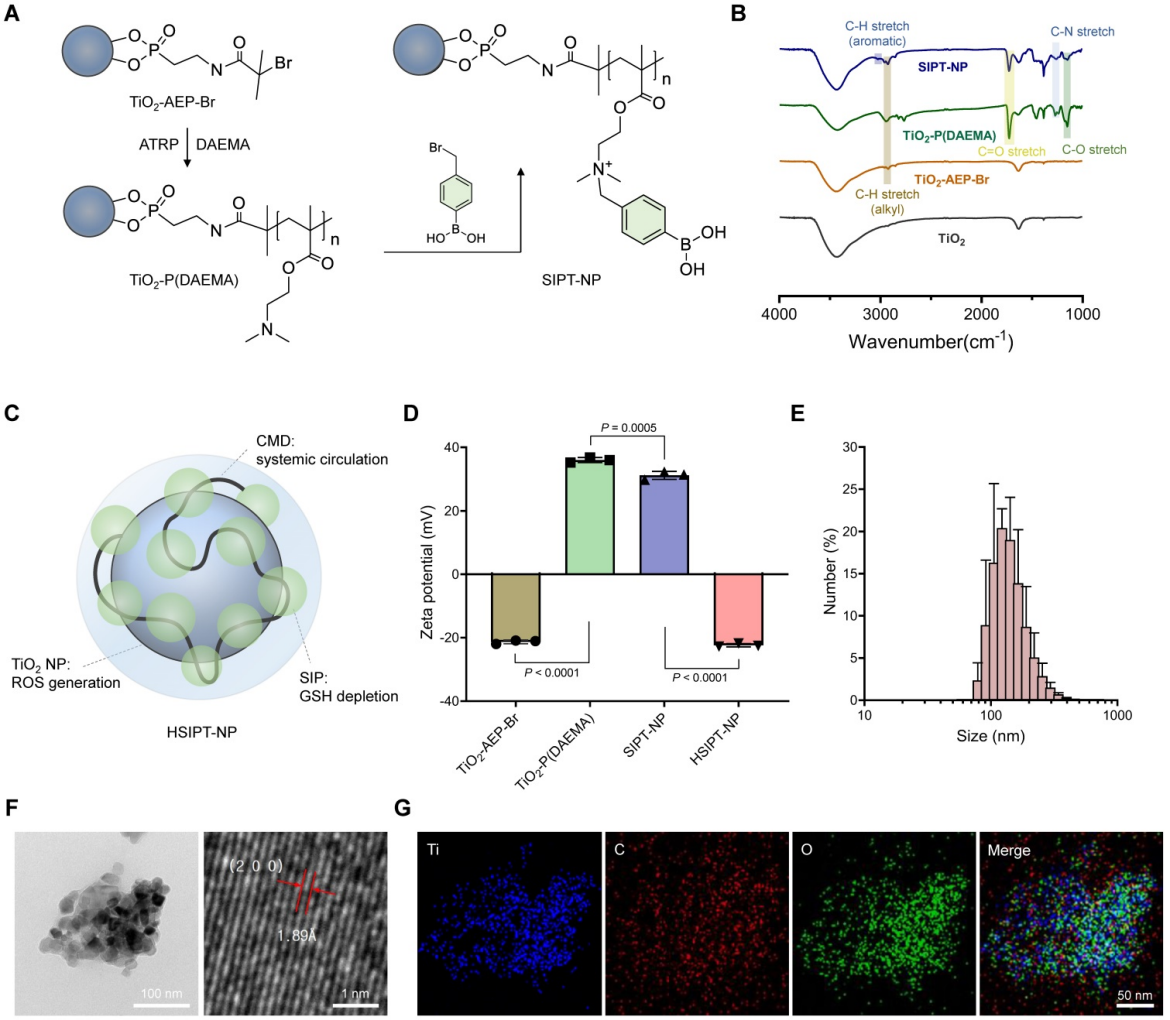
** Characterization of HSIPT-NPs. (A)** Synthetic scheme of SIPT-NPs. **(B)** FTIR spectra of SIPT-NPs. **(C)** Structural information of HSIPT-NPs. **(D)** Zeta potential of HSIPT-NPs. Error bars represent the SD (*n* = 3). P value was analyzed using one-way ANOVA. **(E)** Size distribution of HSIPT-NPs. **(F)** TEM images of HSIPT-NPs (left). High-resolution TEM images show the crystalline structure of HSIPT-NPs (right). **(G)** EDS mapping images of HSIPT-NPs. Blue, red, and green represent titanium, carbon, and oxygen, respectively.

In this study, SIPT-NPs were prepared to achieve on-demand GSH depletion and ROS generation in cancer cells, as shown in Figure 2A. After surface functionalization of TiO_2_ NPs with bromine, 2-(dimethylamino)ethyl methacrylate (DAEMA) was polymerized on the nanoparticular surface. Subsequently, the tertiary amine group of poly(DAEMA)-TiO_2_ NPs was coupled with the bromine group of 4-(bromomethyl)phenylboronic acid (mPBA) to obtain SIPT-NPs. In this study, mPBA was chosen as the self-immolative precursor to release QM because of its high sensitivity to ROS 30. The ester bond of poly(DAEMA) and the aromatic ring of mPBA on the SIPT-NPs were identified using Fourier-transform infrared (FTIR) spectroscopy (Figure 2B). In addition, the molecular weight of SIP was estimated to be 14,244 Da (polydispersity index = 1.44), as measured by gel permeation chromatography (Supplementary method). The chemical structure of the SIP on the surface of the TiO_2_ NPs was also confirmed using ^1^H NMR, suggesting that SIPT-NPs had 53 mPBA molecules per 100 units of poly(DAEMA) (Figure S1). In addition, SIPT-NP consisted of 39.49 wt% of TiO_2_ and 57.97 wt% of SIP, as evaluated by thermogravimetric analysis (Figure S2). Therefore, the prepared SIPT-NPs have the potential to release a large amount of QM at the site of interest.

For prolonged systemic circulation, polyanionic carboxymethyl dextran (CMD) was coated on the surface of SIPT-NPs to prepare HSIPT-NPs, as shown in Figure 2C 31. The decoration of CMD onto the surface of SIPT-NPs decreased the zeta potential value from 31.2 to -21.4 mV (Figure 2D). In addition, HSIPT-NPs had a hydrodynamic size of 143.8 nm and a polydispersity index of 0.189 (Figure 2E and Table S1). Transmission electron microscopy (TEM) analysis showed that HSIPT-NP had an anatase form even after polymerization on the surface of the TiO_2_ NPs (Figure 2F and S3). Energy-dispersive X-ray spectroscopy (EDS) images and FTIR spectra also indicated successful CMD decoration on the surface of the SIPT-NPs (Figure 2G and S4). Owing to the CMD decoration, HSIPT-NPs maintained their hydrodynamic size and zeta potential for at least 24 h (Figure S5). As a control without SIP, we prepared hydrophilic CMD-decorated TiO_2_ NPs (HT-NPs) following a previously described procedure 4. The physicochemical characteristics of HT-NPs were similar to those of HSIPT-NPs, as demonstrated using dynamic light scattering (DLS), TEM, and EDS (Table S1 and Figure S6).

### Depletion of intracellular GSH by HSIPT-NPs

**Figure 3:**
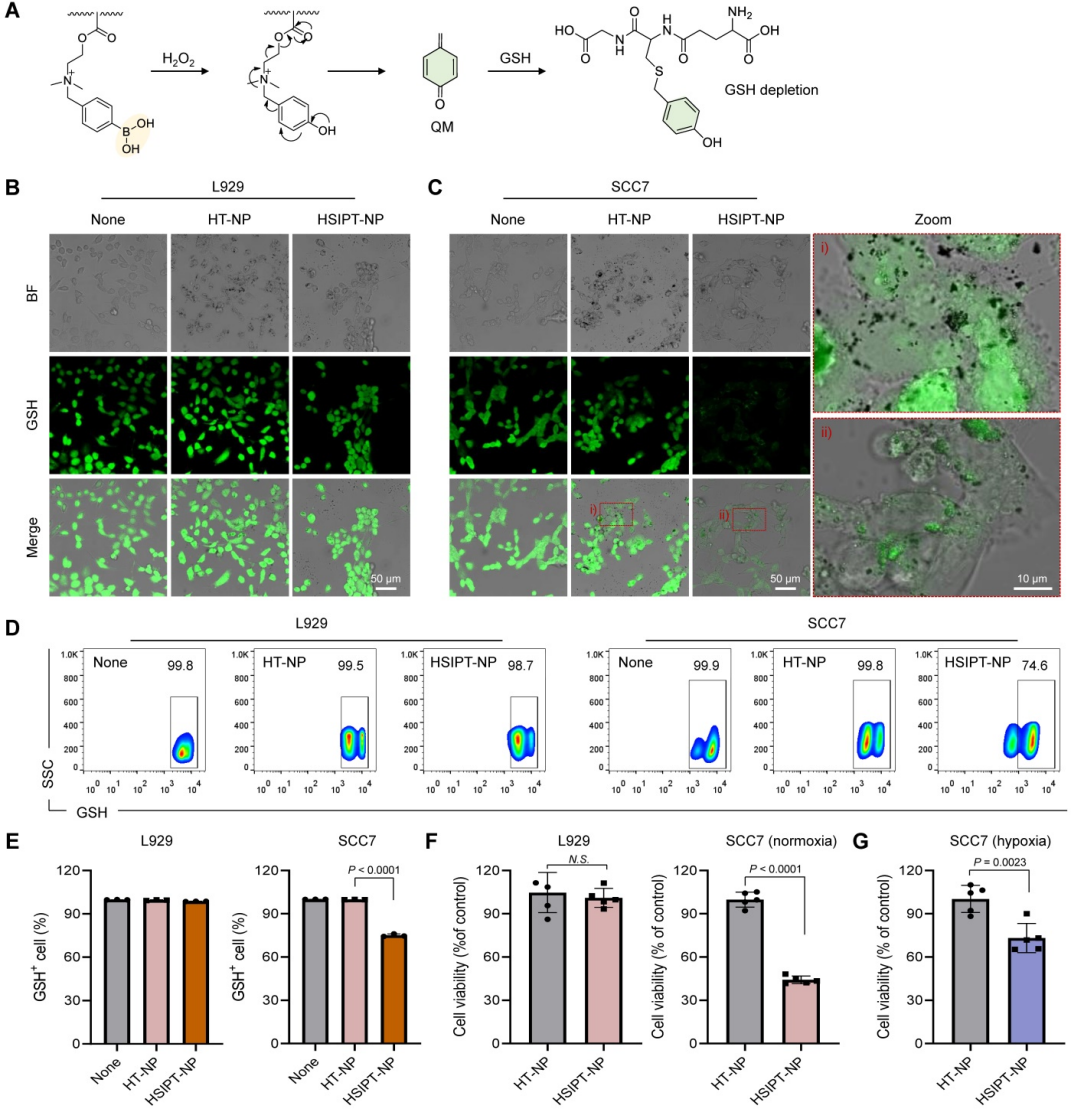
** Intracellular GSH depletion by HSIPT-NPs. (A)** Mechanism of GSH depletion by HSIPT-NPs. **(B)** Confocal microscopy images of GSH (green) in HT-NP- or HSIPT-NP-treated L929 cells. **(C)** Confocal microscopy images of GSH (green) in HT-NP- or HSIPT-NP-treated SCC7 cells. **(D)** Flow cytometry of the GSH marker in TiO_2_ NP-treated L929 or SCC7 cells. **(E)** Quantification of the expression levels of GSH in TiO_2_ NP-treated L929 or SCC7 cells. Error bars represent the SD (*n* = 3). P value was analyzed using one-way ANOVA. **(F)** Cytotoxicity of TiO_2_ NPs against L929, SCC7 (normoxia), or **(G)** SCC7 (hypoxia) cells. Error bars represent the SD (*n* = 5). P values were analyzed using t-test.

As an essential element of the antioxidant defense system, intracellular GSH induces resistance to oxidative stress by scavenging ROS 16. To date, to neutralize the antioxidant defense system of cancer cells, various small-molecule drugs or inorganic materials have been developed that reprogram GSH*^high^* cancer cells into GSH*^low^* phenotype 18-20, 32. However, owing to the off-target toxicity of conventional GSH-depleting drugs, their clinical applications have been limited 33, 34. Therefore, it is essential to evaluate the potential of HSTPT-NPs for on-demand therapeutic action. In this study, when HSTPT-NPs were exposed to H_2_O_2_-rich environments, SIP was rapidly decomposed via electron transfer and depleted GSH by releasing QM (Figure 3A). However, QM is structurally converted into 4-hydroxybenzyl alcohol in aqueous conditions, requiring to deliver QM to the intracellular level for effective depletion of GSH (Figure S7). Thus, to verify the intracellular delivery of HSTPT-NPs, we observed cellular uptake behaviors via confocal microscopy (Figure S8). For both HSTPT-NP-treated L929 and SCC7 cells, the fluorescence signals in the cytosol increased in a time-dependent manner, suggesting that there were no remarkable differences between cell types in the cellular uptake behavior In addition, the cellular uptake behaviors of CMD-coated metal oxide NPs might be dependent on the non-specific interactions 35, 36. Next, to evaluate how effectively HSIPT-NPs can deplete GSH, we investigated the intracellular thiol levels in L929 and SCC7 cells using confocal microscopy (Figure 3B). The intense fluorescence signals of the thiol tracker were observed in both HT-NPs or HSIPT-NPs-treated L929 cells. Owing to the high stability of boric acid at low H_2_O_2_ levels, HSIPT-NPs did not induce GSH depletion in normal cells. Conversely, for SCC7 cells, HSIPT-NPs substantially diminished GSH fluorescence signals (Figure 3C). In addition, when the HSIPT-NP dose increased, intracellular GSH levels gradually decreased only in cancer cells, but there was no change up to a Ti concentration of 2 µg/mL in normal cells (Figure S9). These results suggest that HSIPT-NP-mediated GSH depletion is a safe and effective strategy to specifically disrupt the antioxidant defense system of cancer cells. The intracellular GSH levels in L929 and SCC7 cells were also investigated using flow cytometry, suggesting that intracellular GSH was not depleted in HT-NP- or HSIPT-NP-treated L929 cells (Figure 3D). Interestingly, HT-NP did not affect the GSH levels in cancer cells, but there were superior GSH depletion effects in HSIPT-NP-treated SCC7 cells (Figure 3E). Consistent with the confocal microscopy results, these results suggest that depletion of GSH in cancer cells arose from the ROS-specific degradation of SIP (Figure S10).

To further investigate the on-demand therapeutic actions of HSIPT-NPs on cancer cells, we performed a cell viability assay (Figure 3F). For both HT-NPs- or HSIPT-NPs-treated L929 cells, there was no significant cytotoxicity up to a Ti concentration of 2 µg/mL, suggesting their biocompatible nature in normal cells (Figure S11). No significant cell death was also found in HT-NP-treated SCC7 cells, but more than 55% of cells were died in the presence of HSIPT-NPs (IC_50_ = 1.4 µg/mL), which might be due to their GSH depletion-mediated cytotoxicity (Figure 3F and S11). However, from the perspective of SDT, because traditional sonosensitizers do not effectively work in an oxygen-independent manner, it is important to evaluate the GSH depletion efficacy of HSTPT-NPs under hypoxic conditions 37. Interestingly, under hypoxic conditions, HSIPT-NPs effectively induced GSH-depletion-mediated cytotoxicity in cancer cells (Figure 3G). Overall, these results suggest that QM, released from HSIPT-NPs in response to H_2_O_2_-rich environments, efficiently causes cancer cell death through intracellular GSH depletion.

### HSIPT-NPs as an SDT enhancer *in vitro*

**Figure 4:**
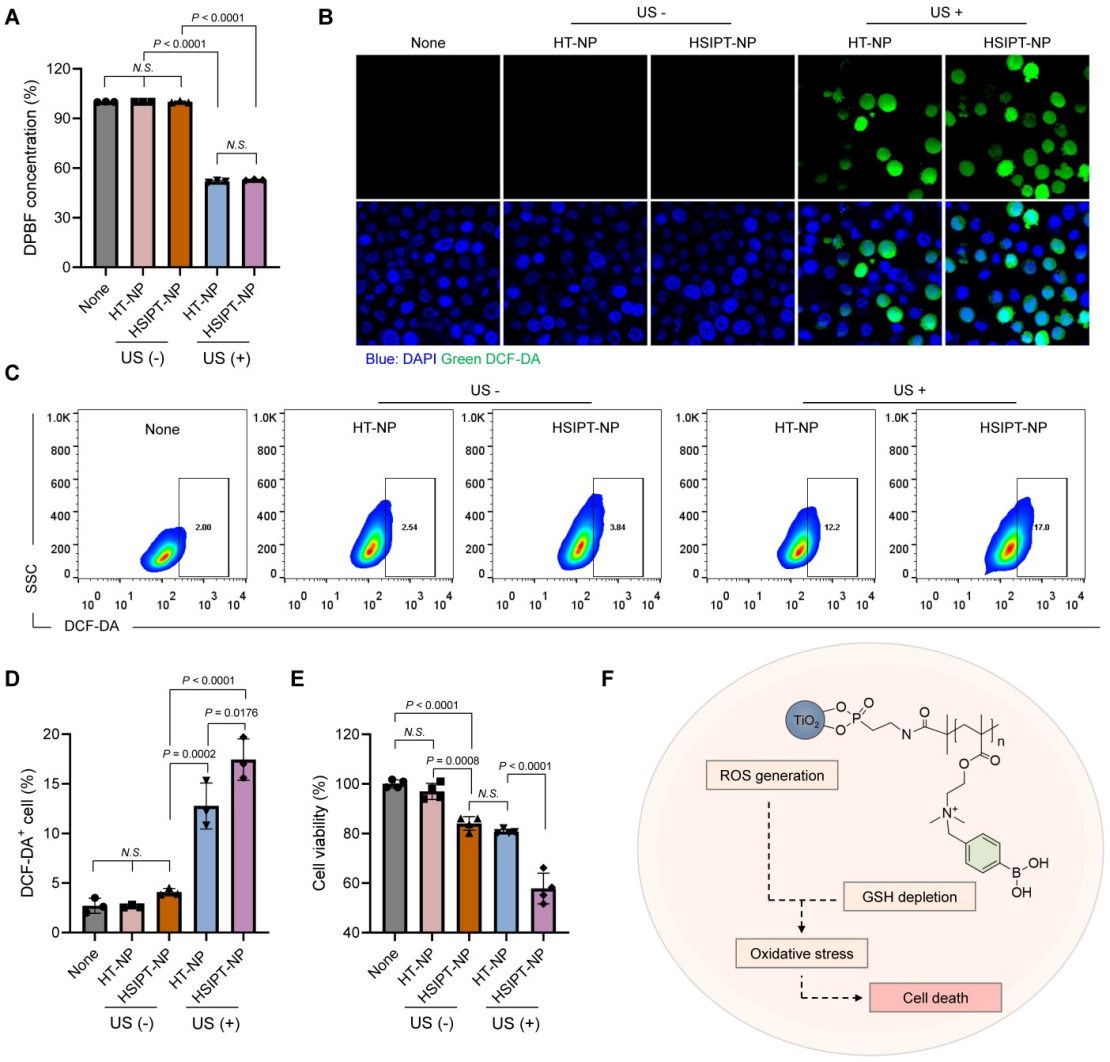
** Characteristics of HSIPT-NPs as an SDT enhancer. (A)** *In vitro* ROS generation by HT-NPs and HSIPT-NPs in the absence or presence of ultrasound. Error bars represent the SD (*n* = 3). P values were analyzed using one-way ANOVA. **(B)** Confocal microscopy images of ROS (green) in HT-NP- or HSIPT-NP-treated SCC7 cells in the absence or presence of ultrasound. **(C)** Flow cytometry of the ROS in TiO_2_ NPs-treated L929 or SCC7 cells in the absence or presence of ultrasound. **(D)** Quantification of the intracellular shown in (C). Error bars represent the SD (*n* = 3). P values were analyzed using one-way ANOVA. **(E)** Cytotoxicity of SDT with HT-NPs and HSIPT NPs in SCC7 cells. Error bars represent the SD (*n* = 5). P values were analyzed using one-way ANOVA. **(F)** Schematic illustration of enhanced SDT by HSIPT-NPs.

For SDT, conventional TiO_2_-based sonosensitizers have poor therapeutic outcomes owing to their low ROS quantum yield. Thus, in this study, we investigated whether a combination strategy with SIP could enhance the therapeutic efficacy of TiO_2_-based sonosensitizers by reprogramming GSH*^high^* cancer cells toward the GSH*^low^* phenotype. First, we assessed the *in vitro* ROS generation efficacy of HSIPT-NPs using 1,3-diphenylisobenzofuran (DPBF) (Figure 4A). On ultrasound irradiation, both HT-NPs and HSIPT-NPs generated a remarkable amount of ROS. Quantitatively, there was no significant difference in the amount of ROS generated by the TiO_2_ NPs. These results suggested that the polymerization process of SIP did not affect the intrinsic ROS quantum yield of TiO_2_ NPs, as confirmed by the crystalline structure of the anatase form (Figure 2E and S6). Next, at the cellular level, we investigated the ROS generation efficacy of HSIPT-NPs using confocal microscopy (Figure 4B). As expected, in the absence of ultrasound, the fluorescence signals of ROS in HT-NP- or HSIPT-NP-treated SCC7 cells were undetectable. Notably, in the presence of ultrasound, HSIPT-NP-treated SCC7 cells exhibited much higher fluorescence signals than the HT-NP-treated SCC7 cells, suggesting that the downregulation of GSH levels in cancer cells provides a favorable environment to ameliorate the efficacy of SDT.

To further verify the synergistic effects of SIP and TiO_2_ NP on SDT, we quantified cellular ROS levels using flow cytometry (Figure 4C-D). We found no significant differences in ROS generation among the non-, HT-NP-, or HSIPT-NP-treated SCC7 cells in the absence of ultrasound. Conversely, in the presence of ultrasound, a strong fluorescence signal was observed in HSIPT-NP-treated SCC7 cells, in which the fluorescence intensity of HSIPT-NP-treated SCC7 cells was 137% higher than that of HT-NP-treated cells. Next, owing to the SIP-mediated GSH depletion, the cytotoxicity of HSIPT-NP-treated SCC7 cells was significantly higher than that of HT-NP-treated cells in the presence of ultrasound even at 0.4 µg/mL Ti concentration (Figure 4E). Moreover, the cytotoxicity observed with HT-NPs in the presence of ultrasound was achievable with the HSIPT-NPs in the absence of ultrasound, suggesting that a substantial amount of cytotoxic ROS generated by TiO_2_-based sonosensitizers is scavenged by GSH in cancer cells (Figure 4E and S12). Taken together, based on reprogramming the GSH*^high^* cancer cells toward the GSH*^low^* phenotype, SIP enhances the therapeutic potential of sonosensitizers in the context of SDT (Figure 4F).

### *In vivo* antitumor efficacy of HSIPT-NPs

**Figure 5:**
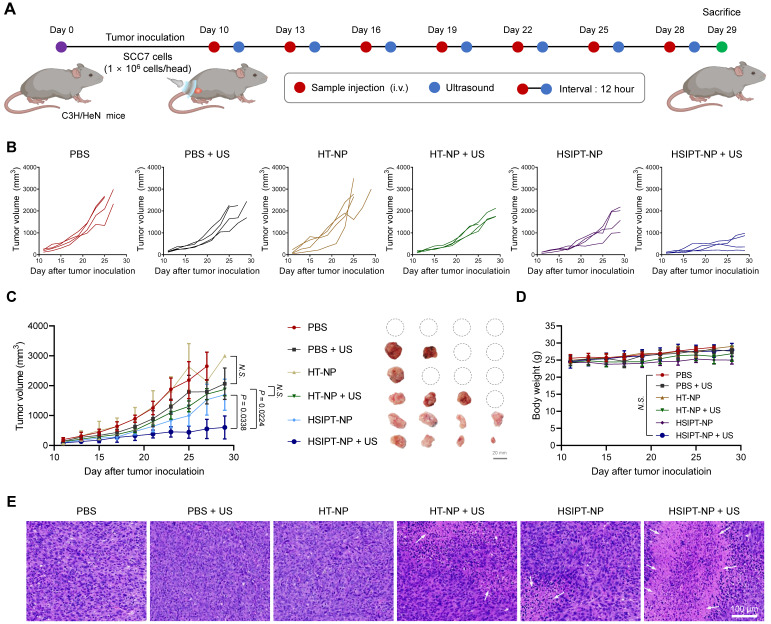
***In vivo* therapeutic efficacy of SDT with HSIPT-NPs. (A)** Schematic illustration of the treatment regimen of SDT with HSIPT-NPs. After intravenous injection of the sample at 2 mg/kg of Ti into SCC7 tumor-bearing mice, ultrasound was irradiated to tumor regions, and changes in tumor volume were monitored as a function of time. **(B)** Individual tumor growth data for each treatment group. **(C)** *In vivo* anti-tumor efficacy of each treatment group (left). Images of tumor tissues treated with SDT on day 29 (right). Error bars represent the SD (*n* = 4). P values were analyzed using one-way ANOVA. **(D)** Changes in body weight by SDT for each treatment group. Error bars represent the SD (*n* = 4). P value was analyzed using one-way ANOVA. **(E)** H&E staining images of tumor tissues for each treatment group.

Having established the feasibility of on-demand GSH depletion and ROS generation *in vitro* (Figure 3 and 4), we next demonstrated the potential of HSIPT-NPs as an SDT enhancer *in vivo* (Figure 5). First, for SDT, it is crucial to evaluate the appropriate ultrasound irradiation timing to maximize the *in vivo* therapeutic efficacy of sonosensitizers. Thus, we investigated the *in vivo* biodistribution of HSIPT-NPs, which are designed for prolonged circulation, in SCC7 tumor-bearing mice (Figure S13). At 12 h post-injection, a prominent fluorescence signal of Flamma 675-labeled HSIPT-NPs was detected in the tumor region, and the surroundings could be demarcated from that. In addition, we verified the prolonged systemic circulation property of HSIPT-NPs by evaluating their whole-body distribution in normal mice (Figure S14). On the basis of these results, we evaluated the antitumor efficacy of HSIPT-NPs as an SDT enhancer by following the treatment regimen (Figure 5A). In this study, the SCC7 tumor-bearing mice were divided into six groups, and mice were treated with PBS, PBS and ultrasound (PBS + US), HT-NP, HT-NP and ultrasound (HT-NP + US), HSIPT-NP, and HSIPT-NP and ultrasound (HSIPT-NP + US). Notably, we conducted an antitumor efficacy test under mild ultrasound power that did not affect tumor growth (Figure 5B). In addition, the PBS or HT-NP treatment groups showed no significant changes in tumor growth inhibition (Figure 5C). The HT-NP + US group showed moderate effects on tumor growth inhibition, and their therapeutic outcomes were comparable to those of the HSIPT-NP monotherapy group. Interestingly, the HSIPT-NP + US group showed significantly enhanced therapeutic outcomes, which might be due to the synergistic effects of ROS generation and GSH depletion. Moreover, compared to the PBS treatment group, the HSIPT-NP + US group exhibited no significant changes in body weight during the treatment (Figure 5D). To verify the site-specific therapeutic action of HSIPT-NPs, we analyzed the histological changes in the tumor and major organs after SDT. It was evident that the HSIPT-NP + US group showed enhanced cell death compared with the other treatment groups (Figure 5E). In addition, no sign of toxicity was found in the HSIPT-NP + US group, suggesting that SDT by HSIPT-NP exhibited tumor-specific therapeutic action and no systemic toxicity (Figure S15). Overall, these results suggest that HSIPT-NP has outstanding potential as a sonosensitizer for enhanced SDT.

## Conclusion

To overcome the limitations of conventional sonosensitizers, we developed HSIPT-NPs that can be employed as potent SDT agents. After intracellular delivery of HSIPT-NPs into hydrogen peroxide-rich cancer cells, they were rapidly degraded through electron transfer, resulting in the reprogramming of GSH*^high^* cancer cells toward GSH*^low^* phenotype by QM-mediated GSH depletion. In addition, owing to the synergistic effects of SIP and TiO_2_ NPs in cancer cells, HSIPT-NPs showed enhanced SDT in tumor-bearing mice. Overall, the SIP-bearing nanosensitizer platform to overcome the low quantum yield of conventional sonosensitizer offers a new sonotherapeutic modality.

## Supplementary Material

Supplementary materials and methods, figures and table.

## Abbreviations

SDT: sonodynamic therapy
ROS: reactive oxygen species
GSH: glutathione
SIP: self-immolative polymer
TiO_2_ NPs: titanium dioxide nanoparticles
HSIPT-NP: hydrophilized SIP-decorated TiO_2_ nanoparticles
TME: tumor microenvironment
QM: 1,4-quinone methide
AEP: 2-aminoethyl dihydrogen phosphate
DAEMA: 2-(dimethylamino)ethyl methacrylate
MTT: methylthiazolyldiphenyl-tetrazolium bromide
DPBF: 1,3-diphenylisobenzofuran
DCF-DA: 2,7-dichlorofluorescin diacetate
FBS: fetal bovine serum
DPBS: Dulbecco's phosphate-buffered saline
TiO_2_-AEP: AEP-decorated TiO_2_ NPs
H&E: hematoxylin and eosin
FTIR: Fourier-transform infrared
CMD: carboxymethyl dextran
TEM: transmission electron microscopy
EDS: energy dispersive X-ray spectroscopy
DLS: dynamic light scattering
