# Supplementary material for: Self-immolative nanosensitizer for glutathione depletion- assisted sonodynamic therapy
Source: Theranostics. 2022 Oct 24;12(17):7465–75. doi: 10.7150/thno.75007 (PMC9691364; doi:10.7150/thno.75007)
Supplement: Supplementary file 1 — Supplementary materials and methods, figures and table. [file thnov12p7465s1.pdf]

(Supplementary data for *Theranostics*)

Self-immolative nanosensitizer for glutathione depletion-assisted sonodynamic therapy

Chan Ho Kim[1,‡], Dong Gil You[1,‡], Pramod Kumar E. K.[1], Kyung Hee Han[1], Wooram Um[1], Jeongjin Lee[2], Jae Ah Lee[1], Jae Min Jung[1], Heegun Kang[1], and Jae Hyung Park[1,2,3]*

[1] School of Chemical Engineering, College of Engineering, Sungkyunkwan University, 2066 Seobu-ro, Jangan-gu, Suwon 16419, Republic of Korea

[2] Department of Health Sciences and Technology, SAIHST, Sungkyunkwan University, 81 Irwon-ro, Gangnam-gu, Seoul, 06351 Republic of Korea

[3] Biomedical Institute for Convergence at SKKU (BICS), Sungkyunkwan University, 2066 Seobu-ro, Jangan-gu, Suwon 16419, Republic of Korea

Corresponding author:* ***Jae Hyung Park*

School of Chemical Engineering, Sungkyunkwan University, 2066 Seobu-ro, Jangan-gu, Suwon 16419, Republic of Korea

E-mail: jhpark1@skku.edu

Materials and methods

Synthesis of model SIP. To estimate the molecular weight of SIP, we synthesized the model SIP by mimicking the radical polymerization of SIPT-NPs. In brief, ethyl α-bromoisobutyrate (25 mg, 0.13 mmol), DAEMA (2.2 mL, 13.2 mmol), and 2,2-bipyridyl (39 mg, 0.25 mmol) were added to a Schlenk flask and mixed with 6 mL of dimethylformamide water/isopropanol (1/1/1, v/v/v). After the mixture solution was purged with N_2 for 30 min, 18 mg of copper bromide was added and the reaction was continued at 25 °C for 24 h under N_2. The reaction mixture was then passed through a silica gel column to remove the copper catalyst. Afterward, the polymer was precipitated in an excess amount of hexane, sequentially washed twice with hexane and diethyl ether, and dried under vacuum for 48 h at 25 ℃. Gel permeation chromatography (Waters GPC systems, Milford, USA) was performed using dimethylformamide containing 15 mM LiBr as eluent.

Characterization of ^{1}H NMR spectra. To calculate the number of mPBA molecules on the SIPT-NPs, we used the ^{1}H NMR (Figure S1) and following equation:

$$\text{The number of mPBA molecules} = \frac{(\text{The integral value of mPBA}'\text{s peak/number of proton})}{(\text{The integral value of poly(DAEMA)}'\text{s peak/number of proton})}$$

Thermogravimetric analysis. To investigate the amounts of TiO_2 and SIP in SIPT-NP, we used a thermogravimetric analyzer (TG/DTA 7300, Seiko instrument, Chiba, Japan). The weight changes were measured under a constant temperature rate of 2 ℃/min.

Cellular uptake behavior. To investigate the cellular uptake of HT-NPs and HSIPT-NPs, SCC7 and L929 cells were seeded in 35-mm confocal dishes at a density of 3×10^5 cells. After 24 h, the cells were incubated with Flamma 675 labeled-TiO_2 NPs for pre-determined time. The cells were

then washed twice with DPBS (pH 7.4) and stained with Hoechst. Afterward, the cells were fixed and imaged using a confocal laser microscope (TCS SP8 HyVolution, Leica Microsystems CMS GmbH, Wetzlar, Germany).

QM release profile. To verify the *in vitro* release profile of QM from the HSIPT-NPs, we performed a high-performance liquid chromatography (HPLC). In brief, HSIPT-NPs (8.7mg/ml of Ti) were dispersed in 100 μM H_2O_2-containing PBS (pH 7.4), and the solution was transferred to a membrane tube (molecular weight cut-off = 3.5 kDa). Afterward, the tube was placed in the release medium (PBS, pH 7.4) and shaken gently in a water bath at a speed of 100 rpm at 37 ℃ for predetermined time points. The amounts of QM released were determined using HPLC (Waters Corp., MA, USA). Due to the QM being structurally converted into 4-hydroxybenzyl alcohol in aqueous conditions, we analyzed the peak of 4-hydroxybenzyl alcohol (mobile phase: methanol/H_2O (8:2, v/v), flow rate: 1 mL/min, UV-vis detection at 240 nm).

***In vivo* biodistribution.** For tumor inoculation, SCC7 cells (1×10^6 cells/head) were injected into the left flank of BALB/c nude mice (5 weeks old, male). After 10 days, 200 µL of Flamma 675 labeled-HSIPT-NP (1 mg/mL) was intravenously injected into the tumor-bearing mice. Fluorescence signals were monitored at predetermined time points using a Lago X system (Spectral Instruments Imaging, Tucson, AZ, USA). At 24 h post-injection, the major organs and tumors were excised from the mice and analyzed by measuring the average fluorescence intensity in the region of interest using embedded software.

To investigate the prolonged systemic circulation properties of HSIPT-NPs, we injected 200 µL of Flamma 675 labeled-SIPT-NPs or HSIPT-NP into normal BALB/c nude mice (5 weeks old,

male) via the tail vein. Fluorescence signals were monitored at predetermined time points using a IVIS and analyzed in an identical manner, as described earlier.

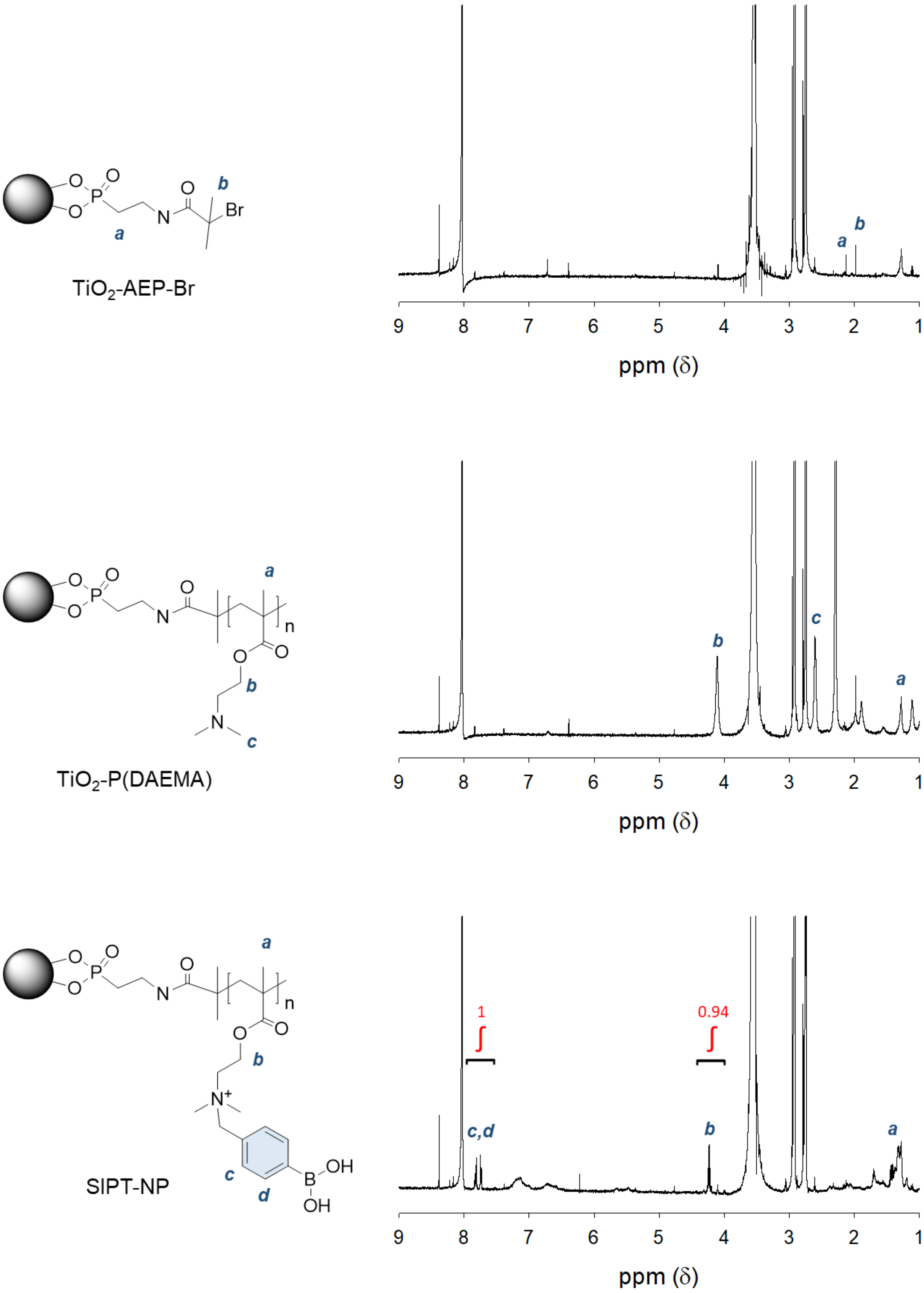

Figure S1. ^{1}H NMR spectra of TiO_2-AEP-Br, TiO_2-P(DAEMA), and SIPT-NP.

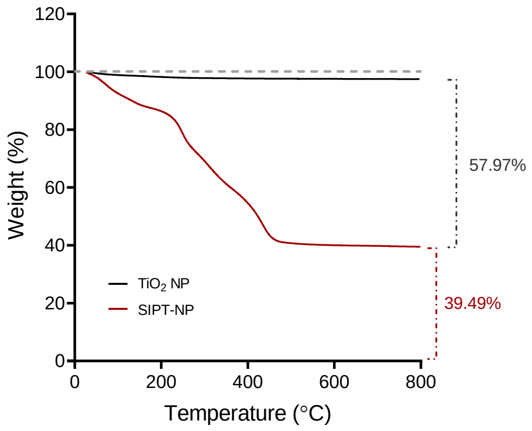

Figure S2. Thermogravimetric curves for TiO_2 NP and SIPT-NP.

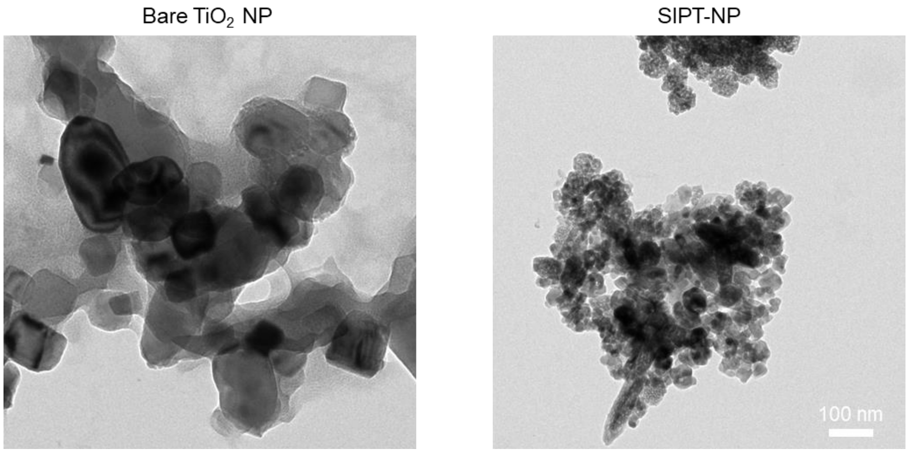

Figure S3. TEM images of bare TiO_2 NP and SIPT-NP.

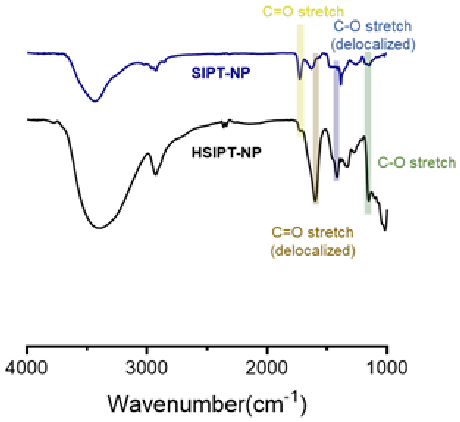

Figure S4. FTIR spectra of SIPT-NP and HSIPT-NP.

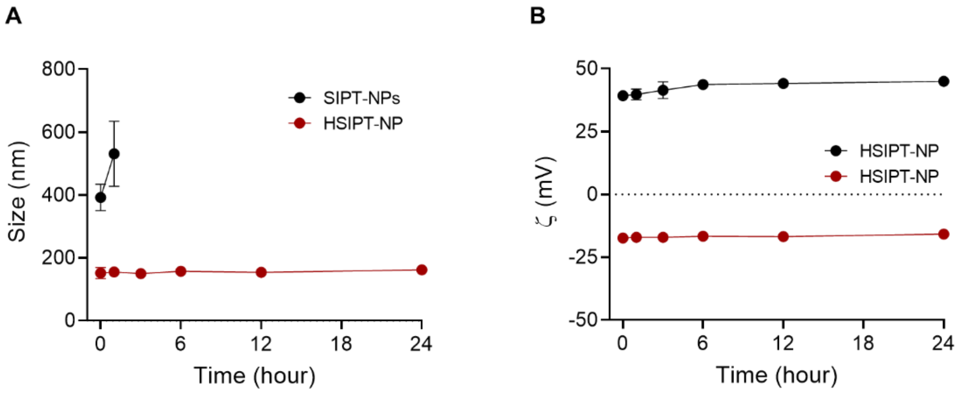

Figure S5. Stability test. (A) Size and (B) zeta potential changes of SIPT-NP and HSIPT-NP as a function of time. Error bars represent the SD (n = 3).

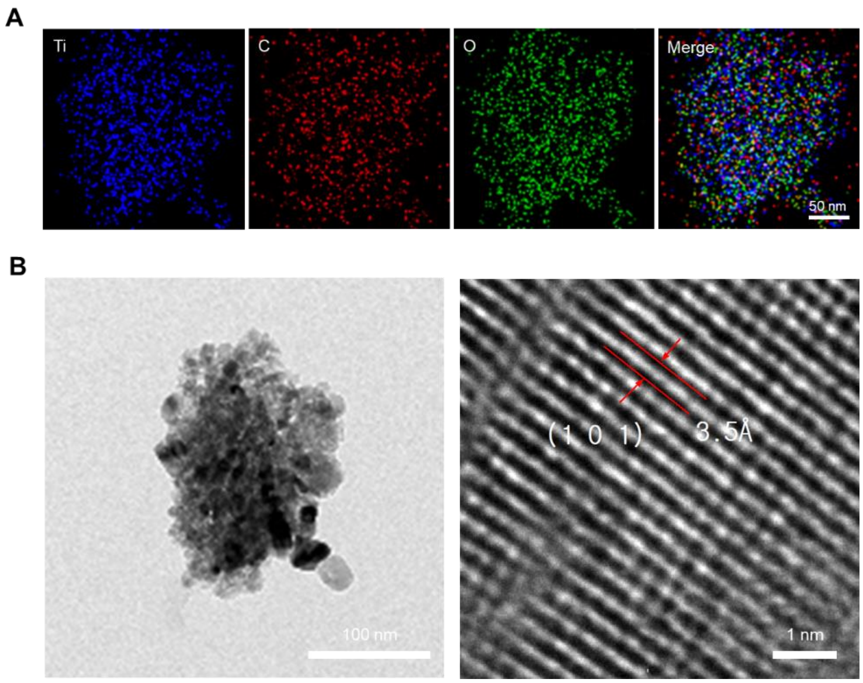

Figure S6. Characterization of HT-NPs. (A) EDS mapping images of HT-NPs. (B) TEM (left) and high-resolution TEM (right) images of HT-NPs.

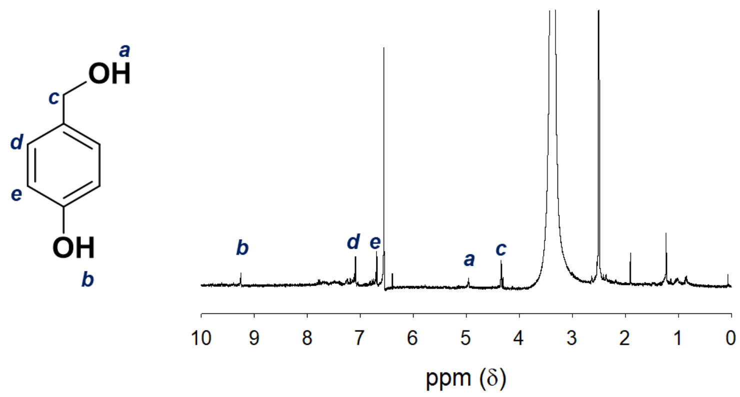

Figure S7. ^{1}H NMR spectrum of 4-hydroxybenzyl alcohol derived from SIPT-NPs.

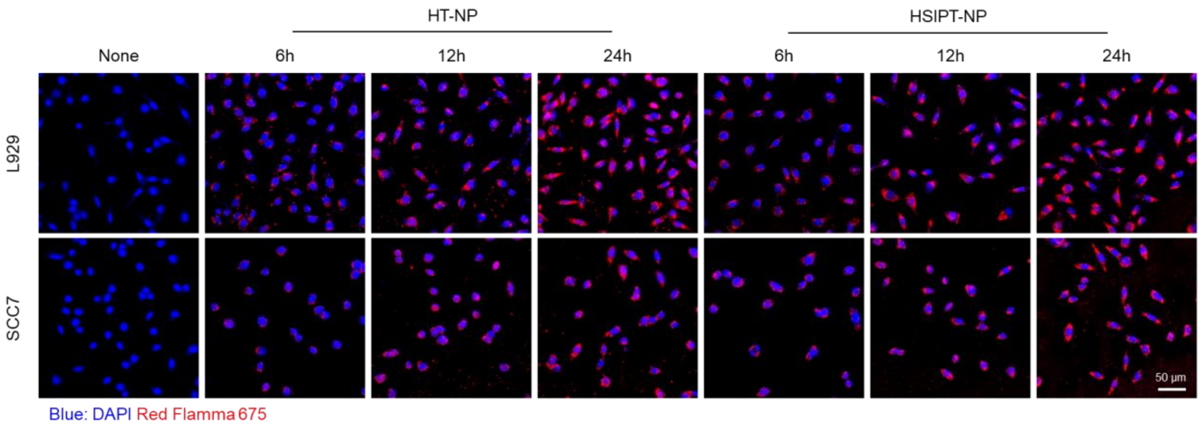

Figure S8. Confocal microscopy images of cellular uptake behaviors of HT-NPs or HSITP-NPs in L929 or SCC7 cells.

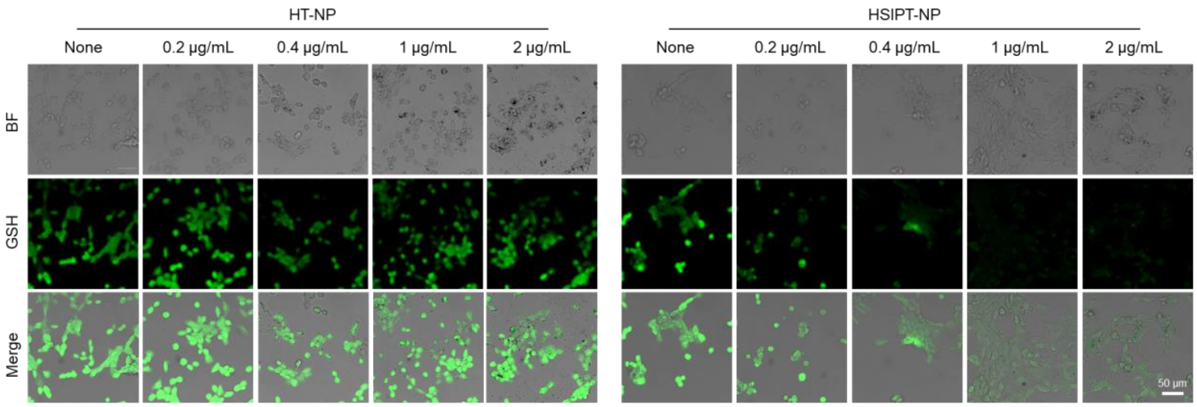

Figure S9. Confocal microscopy images of GSH (green) in SCC7 cells treated with HT-NPs or HSIPT NPs at various concentrations.

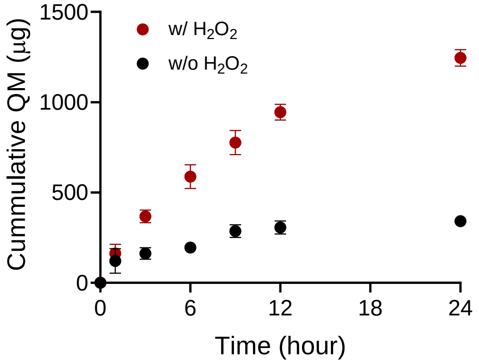

Figure S10. QM release profile of HSIPT-NPs in the presence or absence of 100 μM H_2O_2. Error bars represent the SD (*n* = 3).

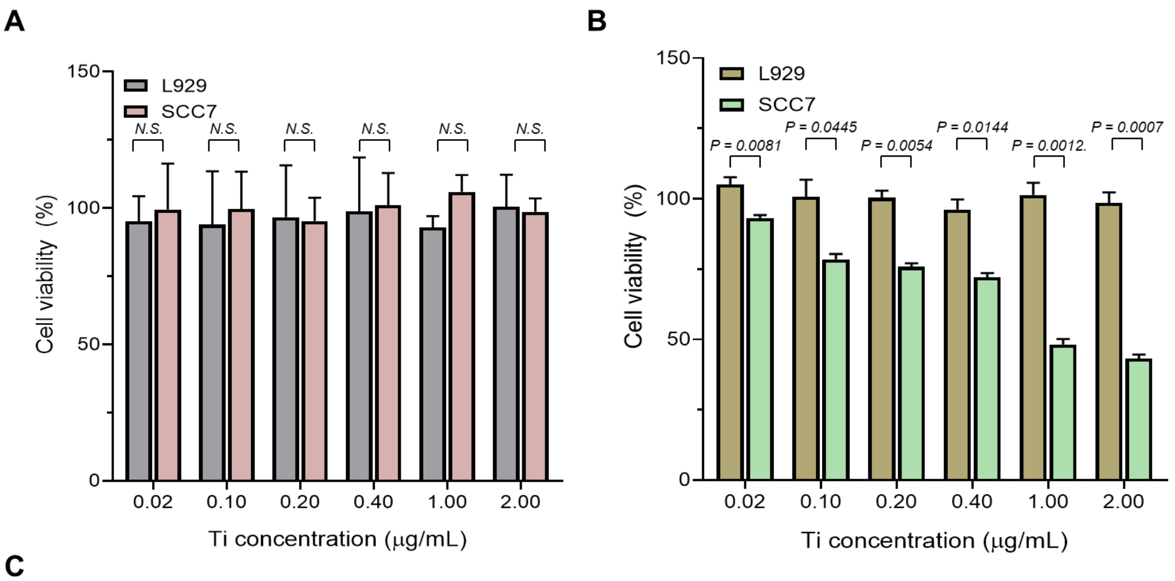

C

Ti concentration (μg/mL)	Mean cell viability of HT-NP-treated L929 cells	Mean cell viability of HT-NP-treated SCC7 cells	Mean cell viability of HSIPT-NP-treated L929 cells	Mean cell viability of HSIPT-NPs-treated SCC7 cells
0.02	95.05121	99.51229	105.1631	93.01561
0.1	93.90756	99.69986	100.7582	78.45111
0.2	96.6107	95.19791	100.2888	75.82169
0.4	98.84597	101.0129	96.17283	72.10353
1	92.9199	105.9526	101.372	48.17174
2	100.4575	98.57437	98.48358	43.13887
IC^{50}	Not applicable	Not applicable	34.3052 μg/mL	1.426019 μg/mL

Figure S11. (A) Cytotoxicity of HT-NPs in L929 or SCC7 cells. (B) Cytotoxicity of HSIPT-NPs in L929 or SCC7 cells. Error bars represent the SD (n = 5). P values were analyzed by two-way ANOVA. (C) Mean cell viabilities and IC_{50} values of HT-NPs or HSIPT-NPs in L929 or SCC7 cells.

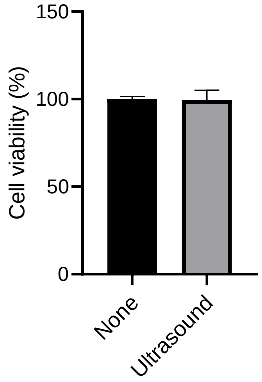

Figure S12. Cytotoxicity of ultrasound irradiation in SCC7 cells.

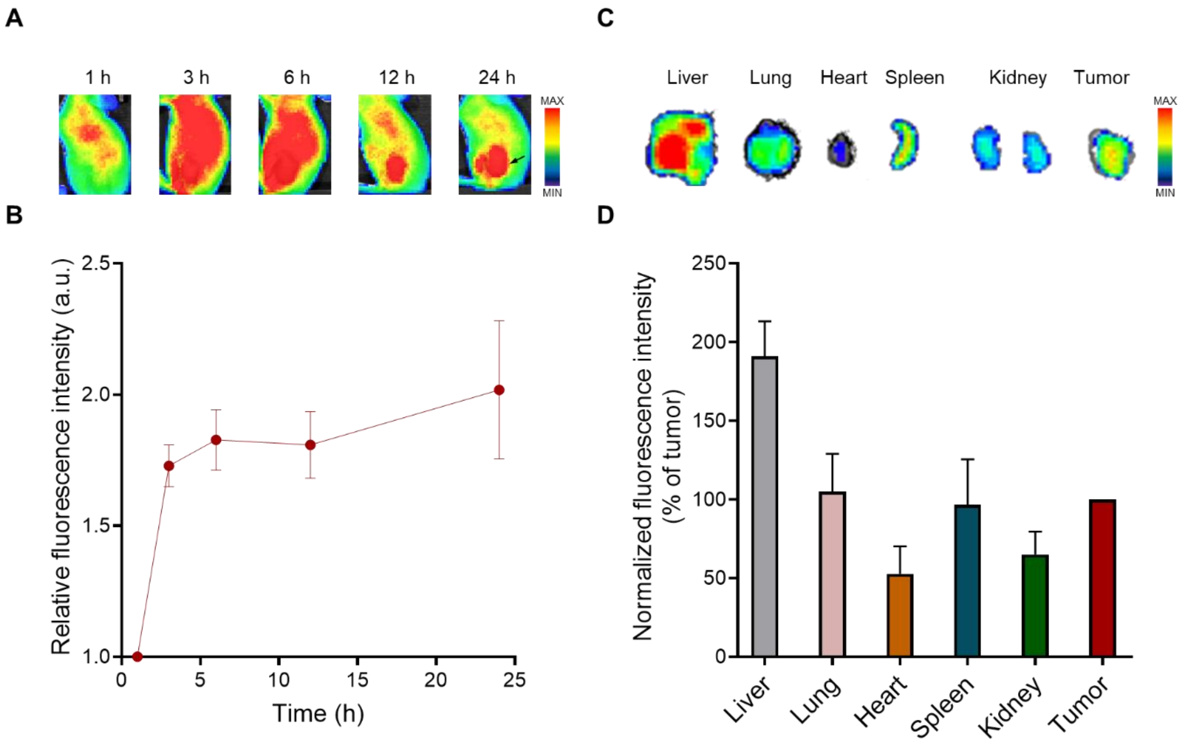

Figure S13. (A) *In vivo* fluorescence images of the Flamma 675-labeled HSIPT-NPs in the tumor-bearing mice. (B) Fluorescence intensity of the HSIPT-NPs in the tumor tissues of the tumor-bearing mice as a function of time. Error bars represent the SD (n = 3). (C) *Ex vivo* organ distribution images of the HSIPT-NPs. (D) Relative fluorescence intensity in the tumor tissues and major organs of the tumor-bearing mice. Error bars represent the SD (n = 3).

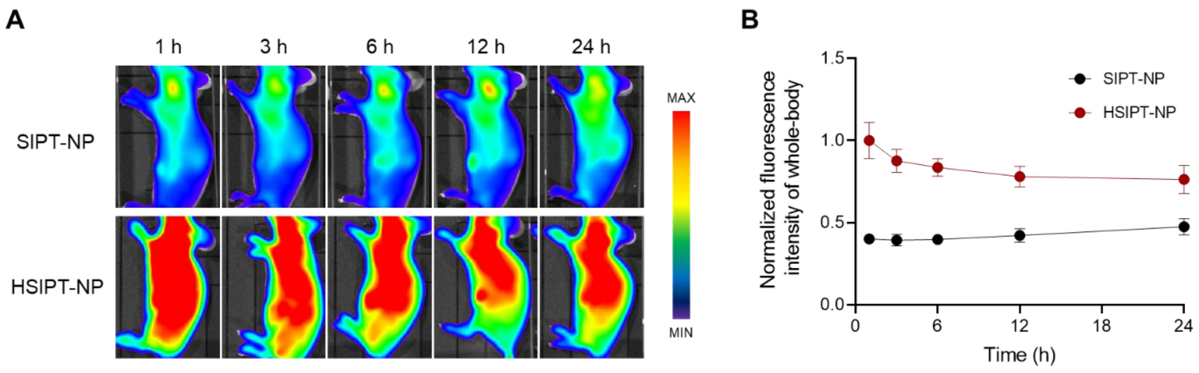

Figure S14. Systemic circulation of HSIPT-NP in normal mice. (A) *In vivo* whole-body fluorescence images of the Flamma 675-labeled SIPT-NPs and HSIPT-NPs in the normal mice (Balb/c nude) as a function of time. (B) Normalized fluorescence intensity of SIPT-NPs and HSIPT-NPs in whole-body fluorescence images of (A). Error bars represent the SD (n = 4).

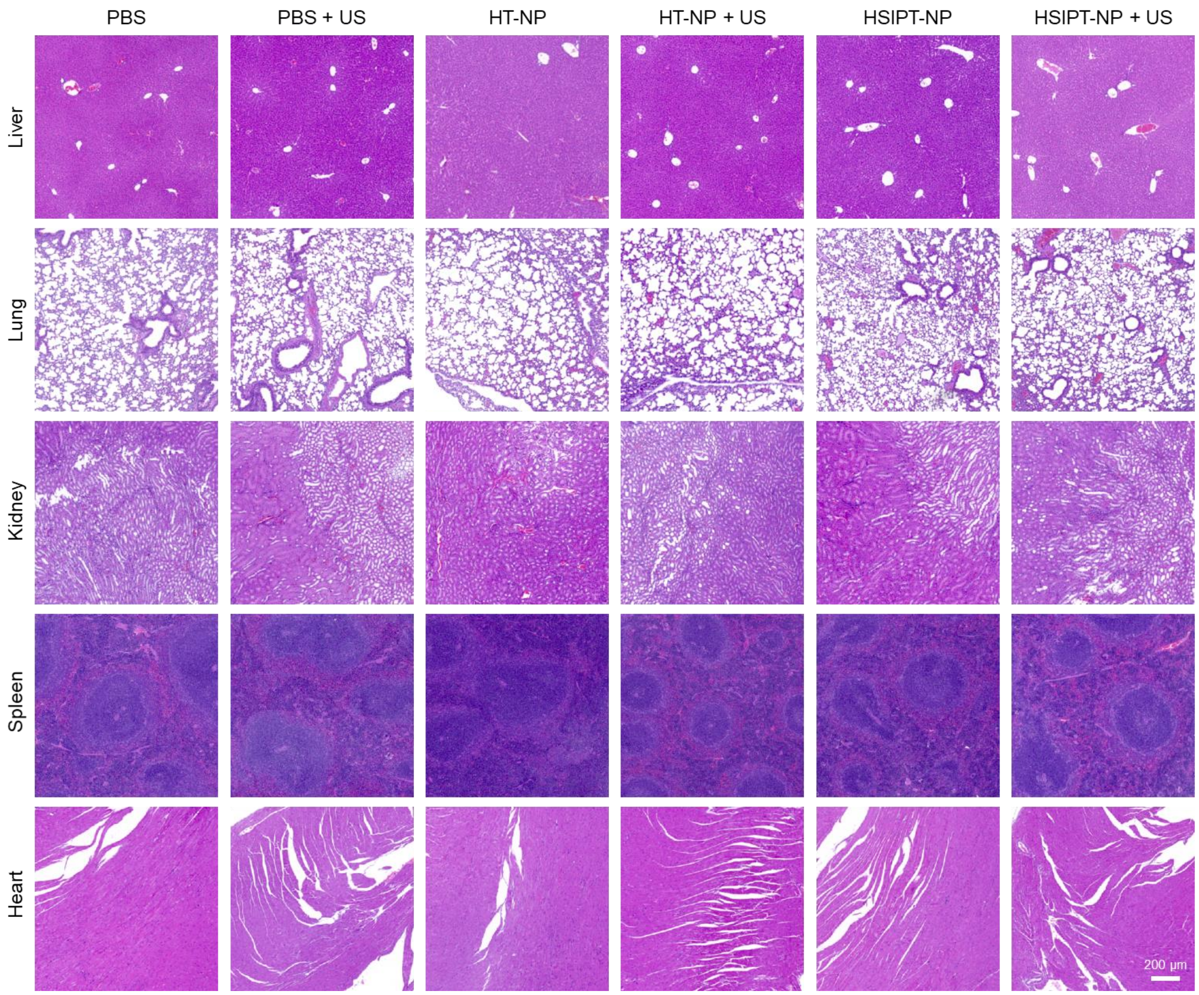

Figure S15. H&E staining images of major organs after treatment.

Table S1. Characterization of the HT-NPs and HSIPT-NPs.

Samples	Size (nm)[a]	Polydispersity index[a]	Zeta potential (mv)[b]	Ti (wt. %)[c]	C (wt. %)[c]	O (wt. %)[c]
HT-NP	173.7 ± 8.17	0.065 ± 0.024	-21.2 ± 0.2	43.52	18.82	37.66
HSIPT-NP	143.8 ± 10.9	0.189 ± 0.007	-16.7 ± 0.2	16.33	62.25	21.42

a) Measured using DLS.

b) Measured using zeta potential analyzer.

c) Measured using TEM-EDS.
